# From the lab to the field and back: the effects of need satisfaction on reconciliation among Germans and Israelis

**DOI:** 10.3389/fpsyg.2024.1243158

**Published:** 2024-10-14

**Authors:** Gali Pesin-Michael, Ruth K. Ditlmann, Nurit Shnabel

**Affiliations:** ^1^The School of Psychological Sciences, Tel Aviv University, Tel Aviv, Israel; ^2^Hertie School of Governance, Berlin, Germany

**Keywords:** the needs-based model, intergroup reconciliation, field study, people-to-people interventions, German–Israeli relations, moral identity

## Abstract

**Introduction:**

Previous lab experiments supported the needs-based model of reconciliation, which posits that discussing historical transgressions enhances the need for acceptance in groups perceived as perpetrators and empowerment in groups perceived as victims. Addressing these needs (e.g., through accepting or empowering messages from outgroup members) increases willingness to reconcile. This study tests this model in a real-world settings.

**Methods:**

Study 1 examined 143 German and Israeli Jewish participants from youth exchange programs, measuring their feelings of acceptance/empowerment, program satisfaction, outgroup attitudes, and keeping in touch with outgroup members. Study 2 examined 293 Israeli Jews, manipulating the salience of the conflict between Israelis and Palestinians in a pre-registered laboratory experiment and measuring responses to accepting versus empowering messages from Germans.

**Results:**

As expected, Study 1 (*N* =143) found Germans’ feelings of acceptance were linked to program satisfaction, positive outgroup attitudes, and keeping in touch. For Israeli Jews, feelings of empowerment were linked to satisfaction and positive attitudes, but keeping in touch was unexpectedly linked to acceptance. This unexpected effect maybe because keeping in touch is done mainly through social networks that focus on the conflict between Israelis and Palestinians, often with a focus on Israel’s transgressions against Palestinians. Consistent with this explanation, Study 2 (*N*=293, pre-registered) showed that Israeli Jews viewed accepting messages from Germans as more conciliatory when presented with transgressions against Palestinians, and empowering messages as more effective when presented with reminders of historical victimization by Nazis.

**Discussion:**

The findings from Study 1 partially support the needs-based model of reconciliation and additionally suggest that reconciliation needs vary with context. Implications for people-to-people peace-building interventions are discussed.

## Introduction

In recent decades there has been a growing recognition that reaching agreements between official group representatives, such as postwar peace or reparation treaties signed by governments, is not enough to achieve genuine intergroup reconciliation (Bar-Siman-Tov, 2004). Rather, grassroots-level peacebuilding initiatives, which are intended to promote trust, mutual acceptance, and cooperation between members of the formerly conflicting groups, are essential as well (Nadler et al., 2008). A common praxis of this peacebuilding approach—which has been adopted by governments and NGOs in various contexts of intergroup conflict (e.g., Maoz, 2004)—is people-to-people interventions, in which ‘ordinary’ people from both sides engage in a *transformative dialogue* to promote more harmonious intergroup relations (Gergen et al., 2001; Gawerc, 2006; Ditlmann et al., 2017b). Such transformation is well illustrated in the words of a Jewish participant of a German Israeli intergroup dialogue program: “The chills that go through my body every time I hear German or meet Germans, are gone. This feeling changed four years ago after I took part in a youth exchange program with Germany and met the new generation of Germans” (Mähler and Kliewe, 2015, p. 66).

But what social psychological processes should take place in an intergroup dialogue to make it transformative? A theoretical framework for addressing this question has been put forward by the needs-based model of reconciliation (Nadler and Shnabel, 2008). The model identifies the psychological needs of members of groups that are perceived as historical or present victims or perpetrators in an intergroup conflict. Note that, like other social psychological models, the model’s focus is on group members’ perceptions (which may be grounded in the historical facts to varying degrees), suggesting that when group members perceive their ingroup as a victim or perpetrator, or learn that their ingroup is perceived as a victim or perpetrator by others, the respective psychological needs arise. The model further proposes that addressing these needs through a mutual dialogue can effectively transform their relations and promote more harmonious intergroup relations. So far, hypotheses derived from the model have been tested almost exclusively in lab experiments (see Shnabel et al., 2023 for a review). Several calls, however, have urged researchers in social psychology to examine their theories outside of the lab to highlight their potential practical value (Cialdini, 2009; Lewis, 2021) and increase their generalizability by describing the associations between the variables of interest in the ‘real world’ (Yarkoni, 2020).

Heeding these calls, Study 1 examined the model in the field for the first time—among German and Israeli Jewish participants of youth exchange programs. Then, because one of Study 1’s hypotheses did not receive empirical support, Study 2 used the advantages of lab experiments (in terms of standardization and controllability) to test a possible explanation for this unexpected result. Specifically, Study 2 tested the hypothesis that the needs of historical victim group members vis-à-vis the historical perpetrator group vary depending on the particular social context that is salient in a given situation. Together, the two studies extend our understanding of how reconciliation processes may unfold in ‘noisy’ real-life conditions.

## The needs-based model of reconciliation

According to the theoretical model that guided our research, members of historically or presently victim and perpetrator groups experience threats to different dimensions of their social identities, resulting in differential motivational states. Members of groups that are perceived as victims suffer from a threat to their agency (i.e., sense of respect, value, voice, and self-determination) and experience an enhanced need for *empowerment*. Members of groups that are perceived as perpetrators suffer from a threat to their moral image, and experience an enhanced need for moral and social *acceptance* (as social exclusion is the sanction imposed upon those who violate the common moral standards; Tavuchis, 1991). The model further argues that the satisfaction of these needs through appropriate messages from the other party can promote reconciliation. Thus, messages from representatives of the perpetrator group that empower the victim group; e.g., by acknowledging its value, competence, achievements, and right to self-determination, would increase victim group members’ willingness to reconcile. Correspondingly, messages from representatives of the victim group that convey moral-social acceptance of the perpetrator group; e.g., by expressing sympathy and brotherhood despite past wrongdoings, would increase perpetrator group members’ willingness to reconcile.

The model’s hypotheses received empirical support in a series of controlled lab experiments, using diverse contexts of intergroup conflict. To illustrate, one study (Shnabel et al., 2009) exposed Israeli-Jewish and German participants to excerpts from two speeches, allegedly made by their outgroup’s representatives at the Berlin Holocaust memorial. The speeches’ main message conveyed either empowerment (e.g., “it is the [Germans/Jews] right to be strong and proud in their country”) or acceptance (e.g., “we should accept the [Jews/Germans] and remember that we are all human beings”). As expected, Jewish participants showed greater readiness to reconcile with Germans following the empowering (vs. accepting) message, whereas German participants showed greater readiness to reconcile with Jews following the accepting (vs. empowering) message. To rule out cultural differences in the importance ascribed to empowerment or acceptance as an alternative explanation, a subsequent study was conducted. In this study, Israeli Jewish and Arab participants were exposed to excerpts from two speeches, allegedly held at the anniversary of the 1956 Kefar Kasem massacre, in which 43 unarmed Arab civilians were killed by the Israeli border patrol. As expected, in this context, Jewish participants showed greater readiness to reconcile with the Arabs following the accepting vs. empowering message from their representative (whereas Arab participants showed the opposite pattern)^1^.

As is typically the case in social psychological lab research (see Ditlmann and Paluck, 2015), these studies have the advantage of high internal validity (i.e., ability to establish causality), but they are limited in terms of ecological validity. For research to have greater ecological validity; that is, to confidently generalize its conclusions to more naturalistic settings, it should be set out in a setting in which the treatment, participants, context, and outcomes are closer to life (Barker, 1968). The goal of Study 1 was to test hypotheses derived from the needs-based model in a setting of a field study, characterized by a higher degree of naturalism (Paluck and Cialdini, 2014; Ditlmann and Paluck, 2015) as compared to the settings in which the model has been tested to date. As we explain in the next section, the exchange programs between German and Israeli Jewish youth were ideal for achieving this goal. Study 2 complemented Study 1 by using a controlled lab experiment in a setting that is less naturalistic but allowed greater causal inference, with the purpose of testing a possible explanation for an unexpected finding of Study 1.

## Youth exchange programs between Germany and Israel: an overview

According to ConAct, the Coordination Center for German-Israeli Youth Exchange, youth exchange programs between Germany and Israel started in the 1950s, when groups of young Germans came to Israel (prior to the establishment of diplomatic relations between the two states) to present a new generation of Germans that wants to atone for past Nazi crimes against the Jews. Since the 1970s, the programs have been officially supported by the governments in Germany and Israel and more than 600,000 people have participated in them so far. Today, the average number of participants is 10,000 youth each year. Seven hundred and ninety-eight different organizations, including municipalities, sports clubs, religious youth movements, scout movements, educational institutions, and peace organizations from Germany and Israel take part in the exchange programs between the countries (Mähler and Kliewe, 2015). These types of exchange programs comprise the core component of the people-to-people peacebuilding process between Germany and Israel, as reflected in the words of Johannes Rau, the President of Germany (1999–2004), in his speech to the Israeli parliament: “I place my hopes in the young people of both countries. If we pass on the memory of the past to the young generation and encourage them to meet, I am convinced we need have no worries about the future of relations between Israel and Germany” (Rau, 2000).

Several characteristics of the German Israeli youth exchange programs made them ideal for testing hypotheses derived from the needs-based model in the field. First, to obtain government funding, besides discussing the importance of intergroup tolerance, protection of human rights, and minorities’ inclusion in multicultural societies as main lessons of the Holocaust, these programs are also obliged to discuss “the history of the expulsion, persecution, and murder of Jews by Germans in Nazi-era Europe” (Mähler et al., 2015, p. 9). Therefore, the victim-perpetrator dyad is likely to be salient in these discussions about the Holocaust—which is the prerequisite for the needs-based model to be applicable.

Second, the possibility of need-satisfaction through the exchange of empowering and accepting messages, as specified by the model, applies to contexts in which members of historically conflicting groups are generally motivated to reconcile (yet psychological barriers still hinder utterly harmonious relations; Shnabel et al., 2023). This is indeed the case in the context of the relations between Germans and Israeli Jews: Over the years, Germany has expressed official acknowledgment of moral responsibility for the Holocaust, and Israelis and Germans have generally positive feelings toward the other country (Hestermann et al., 2022). In this sense, the context of the relations between Germans and Israelis is similar to other contexts in which the historical perpetrator group has accepted responsibility and apologized for the wrongdoing (e.g., the Canadian government’s apology to the First Nations, Métis and Inuit people; Blatz et al., 2009), and different from contexts in which representatives of the historical perpetrator group do not take such responsibility (e.g., Turks’ denial of the Armenian genocide; Bilali, 2013).

Moreover, besides the friendly political atmosphere in general, the motivation to promote reconciliation may be especially high among Germans and Israelis who deliberately chose to participate in youth exchange programs (see Kauff et al., 2021, for an analysis of individuals’ choice to engage in contact opportunities). Therefore, participants of youth exchange programs may be a highly suitable population for testing hypotheses derived from the needs-based model. These participants also expand the subject pool across which the needs-based model has been tested so far. Specifically, participants of youth exchange programs are younger, and their motivation to join the program and participate in the study distinguishes them from people who typically participate in research studies for money or course credit. It can be argued that the types of people who volunteer to take part in a youth exchange program are precisely the ones to whom we want to make sure the needs-based model generalizes because, from a policy perspective, these are the types of people who participate in people-to-people programs in the real world.

Third, although the needs-based model has been tested among Germans and Israelis (Shnabel et al., 2009), the settings in which it was tested simulated intergroup interactions in a relatively artificial way: there was no actual contact between the groups, the communication was unidirectional (participants received messages from their outgroup without conveying their own), the messages were very short (to isolate the ‘active ingredient’ of empowerment or acceptance), and they were conveyed by a single representative of the outgroup. Participants in the youth exchange program between Germany and Israel, by contrast, engage in continuous and dynamic social interactions during and after their participation, which revolve around diverse issues (rather than focusing solely on the historical transgression). In addition to visiting Holocaust museums and memorial sites, they also participate in social activities, learn about other country’s culture, society, and politics, and often continue to interact through social networks after the program has ended (Mähler et al., 2015).

Finally, to date, research within the model’s framework has conceptualized and measured willingness to reconcile as explicit attitudes toward the outgroup. Examining the exchange programs allowed us to test novel outcomes that have not been examined in previous research and go beyond explicit attitudes, namely, behavioral outcomes, such as staying in touch with outgroup members through social networks. Although attitude change is a crucial and important part of reconciliation processes (Staub, 2012), it may or may not co-occur with corresponding behaviors (Paluck, 2009). By examining behaviors such as forming and maintaining cross-group friendships, Study 1 extended the model’s generalizability across measures (see Simons et al., 2017, for the importance of doing so) and addressed the practical needs of program leaders.

Due to the unexpected findings that, among Israelis, the model’s hypotheses were confirmed for one outcome variable but not for the other, we formulated the novel prediction—tested in Study 2—that when the Israeli-Palestinian conflict becomes salient, Israelis experience a heightened need for acceptance (rather than empowerment) in their interactions with Germans. Ultimately, this led us to refine the model’s framework, in line with the logic of self-categorization theory (Turner et al., 1987).

## Beyond the chosen trauma: the fluidity of social identity

The term ‘chosen trauma’ (Volkan, 2021) denotes the shared mental representation of a massive trauma suffered by the group’s ancestors (e.g., the battle of Kosovo for Serbs), which becomes the lens through which members of this group interpret their present-day social reality regardless of how long ago the trauma took place (Szabó et al., 2020)^2^. So far, research within the needs-based model’s framework that examined victim group members’ interactions with members of the perpetrator group responsible for their chosen trauma (e.g., the transatlantic slave trade for African Americans; Ditlmann et al., 2017a,b, the Holocaust for Jews; Shnabel et al., 2009) has focused only on the needs associated with the trauma. This focus stemmed from the assumption that, due to its profound psychological effects, the chosen trauma would produce a chronic or ‘default’ need among members of the victim group vis-à-vis the perpetrator group (in line with findings that, for example, African Americans wish to be empowered by White Americans even in interactions unrelated to slavery or racial relations; Bergsieker et al., 2010)^3^.

Self-categorization theory (Turner et al., 1987), however, emphasizes the flexibility and context sensitivity of people’s social identities, arguing “there is no identity that will determine our behavior irrespective of context” (Reicher, 2004, p. 934; see also Cikara et al., 2022, for the fluid nature of group categories). The theory’s logic suggests that when examining the needs-based model in a non-controlled field setting, multiple victim-perpetrator dyads may become salient—influencing and changing group members’ identity needs. Such changes should emerge even among victim group members interacting with members of the perpetrator group responsible for their chosen trauma.

Study 2 was designed based on the logic of self-categorization theory, in an attempt to explain Study 1’s unexpected finding that Israeli participants of youth exchange programs kept in touch with German program participants to the extent that they felt accepted (rather than empowered) by them. Specifically, Study 2 tested the possibility that in the context of the social networks through which participants kept in touch with outgroup members after the program; namely, a context where Israelis are often viewed as the perpetrators vis-à-vis Palestinians (Seo, 2014), Israelis’ need for moral acceptance would be experienced as more pressing than their need for empowerment. This is in contrast with the context of the exchange program itself, where the historical role of Jews as victims of the Nazis is highly salient—leading to a heightened need for empowerment. Notably, Study 2 was the first to empirically test the fluidity of the needs of historical victim group members even vis-à-vis the perpetrator group responsible for their chosen trauma and even in contexts where perceptions of victimization vs. perpetration are highly contested.

## The present research

The present research consists of two studies. Study 1, a survey among German and Israeli participants of youth exchange groups, was designed with two goals in mind. The first was to meet the practical need of exchange program leaders to know “what works,” by examining whether and how the different types of messages conveyed during discussions about the Holocaust affect program satisfaction. Here, we tested the prediction that Germans’ conveyance of empowering messages (e.g., emphasizing the contribution of the Jewish people to humanity) would be associated with Israelis’ satisfaction with the program (*H*_1a_), whereas Israelis’ conveyance of accepting messages (i.e., emphasizing the common humanity of Germans and Jews) would be associated with Germans’ satisfaction with these discussions (*H*_1b_).

The second goal was to examine the generalizability of the needs-based model to a novel context, characterized by higher degrees of naturalism than the contexts examined in previous research. To address this goal, we tested the predictions that the empowerment of their ingroup by Germans during discussions about the Holocaust would be associated with Israelis’ positive attitudes toward Germans (*H*_2a_) and keeping in touch with Germans following the program (*H*_3a_). Correspondingly, the acceptance of their ingroup by Israelis would be associated with Germans’ positive attitudes toward Israelis (*H*_2b_) and keeping in touch with Israelis following the program (*H*_3b_).

Study 2 was a pre-registered experiment designed to test a possible explanation for the unexpected finding that, inconsistent with *H*_3a_, Israelis’ keeping in touch with Germans following the program was associated with the feeling that their ingroup was accepted, rather than empowered, by Germans. We theorized that although the victim group’s chosen trauma is central to group members’ identity (Szabó et al., 2020), victim group members’ needs vis-à-vis the perpetrator group are not chronic but rather determined by the social context. Using an experimental design, Study 2 tested whether (*H*_4_) Israeli Jewish participants would perceive empowering (vs. accepting) messages from Germans as more conciliatory when the Holocaust is salient, yet the opposite pattern would emerge when the Israeli-Palestinian conflict is salient. By integrating an applied field study (Study 1) and a basic lab experiment (Study 2), the present research contributed to theory building in the field (Lewis, 2021).

### Study 1

Study 1 was a correlational field study of participants in German Israeli youth exchange programs. It was originally planned, in collaboration with Coordination Center for German-Israeli Youth Exchange (ConAct), as a large-scale study that would use qualitative content analysis as the primary research method (to examine questions that are of direct interest to the program leaders). However, the cancellation of programs because of the COVID-19 pandemic led to a change in our plans, resulting in the decision to focus on testing only the hypotheses derived from the needs-based model while using quantitative analyses.

### Method

#### Participants

The Coordination Center for German-Israeli Youth Exchange, which works in collaboration with the Israel Youth Exchange Authority, distributed the link to the online survey about “youth exchange programs between Israel and Germany” to program leaders, who were asked to send it to their program alumni. The data was collected between November 2020 to January 2021. Overall, 69 Germans and 91 Israelis completed the survey in exchange for a small reimbursement. Seventeen participants who indicated that their program did not include any discussions, neither structured nor informal, about the Holocaust were not included in the analysis. Thus, our final sample included 64 Germans (42 women, 22 men) and 79 Israelis (38 women, 37 men, and four participants who did not specify their gender). A sensitivity analysis for a 5% level of significance and a power of 80% revealed that our sample sizes were sufficient to detect correlations of *r* = 0.27 for the Israeli sample and correlations of *r* = 0.30 for the German sample. The observed correlations for both samples (*r*s > 0.35) exceeded this minimum value.

All German participants (*M*_age_ = 20.4, *SD* = 9.4, range = 14 to 66) were born in Germany and came from 13 different states of Germany (see Table 1). Among Israeli participants (*M*_age_ = 18.9, *SD* = 7.2, range = 14 to 60), 74 participants were born in Israel, one in Australia, one in Germany and three did not specify their country of birth. The Israeli participants came from 28 different cities of Israel (see Table 1). Of the German sample, 36 were Christians, 26 were Atheists, and two did not specify their religion. Most of German participants described their political orientation as center (35.9%) or left (31.2%) and the rest did not indicate their political orientation (32.8%). Of the Israeli sample, 72 were Jewish, four were Atheists, and three did not specify their religion. Most Israeli participants described their political orientation as left (34.2%) or center (31.2%) and the rest as right (11.4%) or did not indicate their political orientation (24%).

**Table 1:** German participants’ states of residence and the israeli participants’ regions of residence (Study 1).

States of Germany	Number German of participants	Regions of Israel	Number of Israeli participants
Lower Saxony	12	Center region	30
Berlin	8	Tel Aviv region	21
Saxony	7	The Negev and South	13
Baden-Württemberg	7	Jerusalem region	7
North Rhine-Westphalia	6	Haifa region	4
Brandenburg	6	North region	1
Bavaria	4		
Hamburg	3		
Saxony-Anhalt	3		
Hesse	2		
Mecklenburg-Vorpommern	2		
Schleswig-Holstein	1		
Rhineland-Palatinate	1		
Total	62	Total	76

Most of the participants took part in an exchange program during the years 2019 (43.9%) or 2018 (40.1%), and the rest during the year 2017 (9.8%) or between the years 2010–2016 (6.2%). Both German and Israeli samples came from diverse programs (e.g., youth movements, summer camps, diplomacy-oriented programs, and exchange programs between cities). More than 30% of Jewish and German participants answered the question “in which program did you participate?” in a way that does not allow identifying the program (with answers such as “from school” or “youth exchange”). Nonetheless, we were able to identify nine different German programs and seven different Israeli programs.

All the participants (100%) reported having social activities (beach, parties, sight-seeing) in their programs, the majority of them further reported that their program included acquaintance with contemporary culture and lifestyle (e.g., food, habits) in Germany and Israel (97.84%), homestay with a German or Israeli family (78.26%), and discussions about the Israeli-Palestinian conflict (75.71%).

#### Measurement instruments

##### Empowerment and acceptance in discussions about the Holocaust

Participants who indicated that their program included discussions about the Holocaust were asked “How prominent were the following messages from the outgroup (i.e., “the Israeli group” for German participants, “the German group” for Israeli participants) during Holocaust-related discussions?.” Note that participants’ retrospective evaluations may not be accurate in reflecting their impressions when experiencing the discussions in real-time (Aldrovandi et al., 2015). Nevertheless, when assessing the past, people do not attribute equal weight to each moment of the experience but rather tend to overweight “pick” moments (i.e., moments in which the intensity of the experience was highest; Fredrickson, 2000). Therefore, by relying on participants’ retrospective accounts we likely captured the messages that had special psychological significance for them, which was consistent with Study 1’s purposes. Another concern related to relying on participants’ retrospective memories is that such memories might be influenced by either a positivity bias (people’s tendency to hold “a rosy view” of the past; Adler and Pansky, 2020) or a negativity bias (the tendency to give greater weight to negative events; Rozin and Royzman, 2001). The absence of ceiling and floor effects in the main outcome variables (i.e., program satisfaction and conciliatory tendencies see below), however, does not lend support to the presence of extreme memory biases.

Using a 5-point scale (1 = *this message was never conveyed* to 5 = *this message was extremely prominent*) adapted from Shnabel et al.’s (2009), three items assessed the prominence of messages that empower the historical victim group (“It is vital that Jews have a homeland in which they can feel safe,” “It is important to remember that Jews have made and continue to make immense cultural contributions to humanity” and “Jews should have their own state where they can determine their own fate”;^4^ α = 0.679). Three additional items, adapted from Shnabel et al.’s (2009), assessed the prominence of messages that convey mutual intergroup acceptance (“Despite the past, there is now a strong warm connection between Germans and Israelis,” “We should move on from the past and focus our energy on the current special relationship between Germany and Israel” and “We should be committed to each other as human beings regardless of our group affiliation”; α = 0.601).

Possibly reflecting the general positivity of both message types, the scales measuring empowerment and acceptance were moderately positively correlated (*r* = 0.49, *r_Germans_* = 0.50 and *r_Israelis_* = 0.42; *p*s < 0.05). We therefore created residualized versions of these variables for the testing of our hypotheses. Thus, when assessing the effects of empowerment on the outcome variables, we used the residuals of a regression in which acceptance predicted empowerment as our predictor. When assessing the effects of acceptance on these outcomes, we used the residuals of a regression in which empowerment predicted acceptance as our predictor. This allowed us to examine only the portion of the variance that is theoretically relevant; i.e., ‘pure’ empowerment that does not overlap with acceptance and ‘pure’ acceptance that does not overlap with empowerment (for the reasoning behind using this analytic approach; e.g., avoiding multicollinearity, see Hässler et al. (2021), who employed the same analytic approach).

Note that previous lab experiments measured not only the empowerment of the victim group, but also the empowerment of the perpetrator group; for example, participants in Shnabel et al.’s (2009) studies were asked to what extent the speech of the Jewish representative conveyed the message that Germans have the right to feel strong and safe in their homeland. However, because the present study was conducted within a naturalistic setting, asking about Germans’ empowerment might seem awkward or even offensive to participants. We therefore did not assess the prominence of messages that empower the German group in discussions about the Holocaust within the program. Notably, unlike the empowerment of one group, mutual acceptance — by definition — entails symmetry between the groups. Therefore, we were able to measure acceptance for both Germans and Jews (assessing the extent to which Germans felt accepted by Israelis and vice versa).

##### Program satisfaction

Using 5-point scales (1 = *dissatisfied* to 5 = *extremely satisfied*), four items asked participants about the extent to which they were satisfied with different aspects of the program (e.g., “The interaction and dialogue between the German and Israeli group”; “The content and activities of the program,” *α* = 0.705).

##### Reconciliatory orientation

Reconciliation is a multifaceted construct (for discussion see Nadler, 2012), which can broadly be defined as “a changed psychological orientation toward the other” (Staub, 2006, p. 868). To capture this multifaceted construct, we assessed participants’ reconciliatory orientation toward the outgroup by using 13 items, which included, in line with the notion that ‘the personal is political,’ both group-level items and individual-level items (i.e., maintaining personal relationships with outgroup members). Specifically, participants were asked about their explicit attitudes toward the outgroup and their relations with it (adapted from Shnabel et al., 2009; Hagemann and Nathanson, 2015), subtler behaviors that reflect engagement and interest in the outgroup (adapted from Ditlmann et al., 2017a) and the extent to which they stayed in touch with outgroup members whom they met in the program (adapted from Hässler et al., 2021). To examine the underlying factorial structure of the 13 items, we conducted an exploratory factor analysis (EFA) which yielded a two-factor solution (see Supplemental materials for the correlations between all items, and the results of the EFA). After removing three items that displayed lower loadings than the other items and could not be meaningfully interpreted, we computed two subscales of reconciliatory orientation: positive attitudes toward the outgroup, calculated as the average of seven items, and keeping in touch with the outgroup members, calculated as the average of three items.

The first subscale, consisting of seven 5-point items (1 = *disagree* to 5 = *strongly agree*), captured participants’ positive attitudes toward the outgroup on various dimensions such as liking, interest, and policy support (e.g., “The program bonded me to Israelis/Germans”; “The government should fund programs of exchange between Germans and Israelis”). Items were averaged to obtain the measure of positive attitudes toward the outgroup. This positive attitude subscale is in line with Čehajić-Clancy et al. (2016) conceptualization of reconciliation as a process of emotional regulation involving positive affective change.

The second subscale captured keeping in touch with the outgroup members following the program. Willingness to engage in contact with outgroup members has been conceptualized in the intergroup relations literature as a manifestation of reconciliatory tendencies (e.g., among members of conflicting groups in the aftermath of the Liberian civil wars; Mazziotta et al., 2014). The second subscale consisted of three items: (1) the number, between zero to 10, of outgroup members with whom participants stayed in touch following program; (2) the frequency of contact with outgroup members with whom they stayed in touch, on a 6-point scale (0 = *not at all* to 5 = *daily*), and (3) the quality of contact with outgroup members with whom they stayed in touch on a 4-point scale (1 = *slightly close* to 4 = *extremely close*). The three items were standardized because they were measured on different scales and then averaged to obtain the measure of keeping in touch with outgroup members.

Note that some definitions of reconciliation include structural changes such as equal political rights and allocations of state resources, such as budgets and lands (Nadler, 2012). Since Germans and Israeli-Jews belong to different societies (as compared to contexts where historically conflicting groups share the same society, such as Canada or South Africa) structural changes are complex and were not measured.

### Results

Tables 2, 3 present means, standard deviations, and correlations between the study’s main variables in the Israeli and German samples (respectively). The correlations are presented for both the raw and residualized scores of empowerment and acceptance. Testing for differences between the groups revealed that Germans and Israelis had similar levels of perceptions of empowerment and acceptance, *p*s > 0.85, yet Germans’ program satisfaction was significantly higher than that of Israelis, *t*(142) = 2.32, *p* = 0.02, *d* = 0.39, and so was their reconciliatory orientation, both in terms of their positive attitudes toward the outgroup, *t*(159) = 5.01, *p* < 0.001, *d* = 0.80, and the extent to which they kept in touch with outgroup members following the program, *t*(160) = 3.57, *p* < 0.001, *d* = 0.57. These results are consistent with the general finding that members of historical perpetrator groups are more motivated to reconcile than members of historical victim groups (Wohl et al., 2011).

**Table 2:** Means, standard deviations, and correlations between Study 1 main variables among israeli participants.

	Mean (*SD*)	(1)	(2)	(3)	(4)	(5)
(1) Empowerment	3.4 (1.1)	--	−0.30**	0.31**	0.23*	0.07
(2) Acceptance	3.7 (0.9)	0.58**	--	0.20	0.29*	0.33**
(3) Program satisfaction	3.6 (0.9)	0.41**	0.37**	--	0.51**	0.34**
(4) Positive attitudes toward the outgroup	3.7 (0.7)	0.35**	0.42**	0.51**	--	0.57**
(5) Keeping in touch with outgroup members	−0.2 (1.0)	0.21	0.38**	0.34**	0.57**	--

*n* = 79. Correlations with raw scores of empowerment and acceptance are reported below the diagonal and correlations with residualized scores of empowerment and acceptance are reported above the diagonal. For keeping in touch with outgroup members mean and standard deviation are based on standardized scores. *p < 0.05. **p < 0.01.

**Table 3:** Means, standard deviations, and correlations between Study 1 main variables among german participants.

	Mean (*SD*)	(1)	(2)	(3)	(4)	(5)
(1) Empowerment	3.5 (0.8)	--	−0.62**	−0.29*	−0.20	−0.20
(2) Acceptance	3.7 (0.9)	0.25*	--	0.52**	0.38**	0.37**
(3) Program satisfaction	3.7 (1.0)	−0.02	0.50**	--	0.48*	0.16
(4) Positive attitudes toward the outgroup	4.2 (0.5)	0.00	0.37**	0.48*	--	0.37**
(5) Keeping in touch with outgroup members	0.3 (0.6)	−0.01	0.35**	0.16	0.37**	--

*n* = 64. Correlations with raw scores of empowerment and acceptance are reported below the diagonal and correlations with residualized scores of empowerment and acceptance are reported above the diagonal. For keeping in touch with outgroup members mean and standard deviation are based on standardized scores. *p < 0.05, **p < 0.01.

#### Program satisfaction

**Figure 1:**
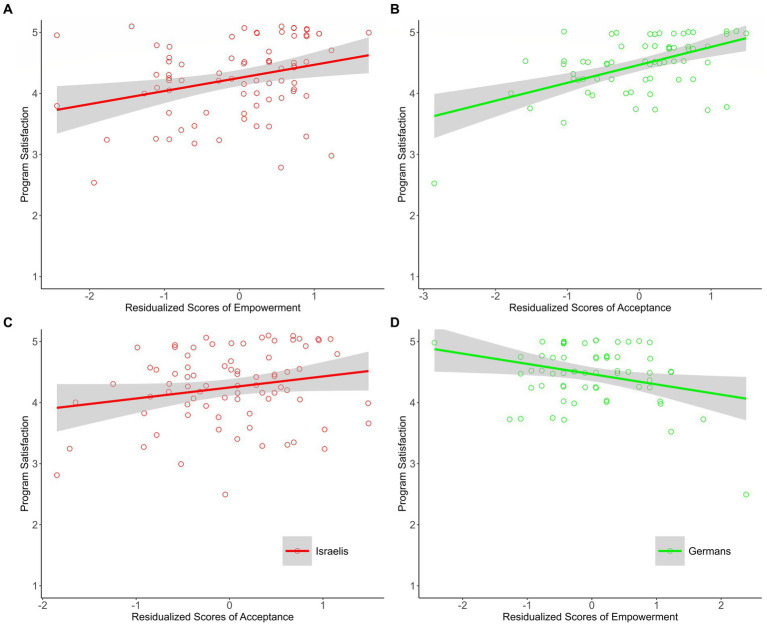
The associations between residualized scores of empowerment and acceptance and program satisfaction among Israeli and German participants of Study 1. Israeli group (*n* = 79), German group (*n* = 64). The plots for the needs about which we had explicit predictions appear in the upper part of figure **(A,B)**; the plots for the ‘other’ needs appear in the lower part of figure **(C,D)**. Scatterplots and regression lines with 95% confidence bands for the association between residualized scores of empowerment and acceptance and program satisfaction. Raw data points are jittered.

Our analysis (using the residualized scores) revealed that, in line with predictions, prominent messages of empowerment of the victim group within discussions about the Holocaust were associated with Israelis’ program satisfaction (see Figure 1A), whereas prominent messages of acceptance were associated with Germans’ program satisfaction (see Figure 1B). As for the ‘other’ need, prominent messages of acceptance were not associated with Israelis’ program satisfaction (see Figure 1C), whereas prominent messages of empowerment of the victim group were negatively associated with Germans’ program satisfaction (see Figure 1D).

#### Reconciliatory orientation

##### Positive attitudes toward the outgroup

**Figure 2:**
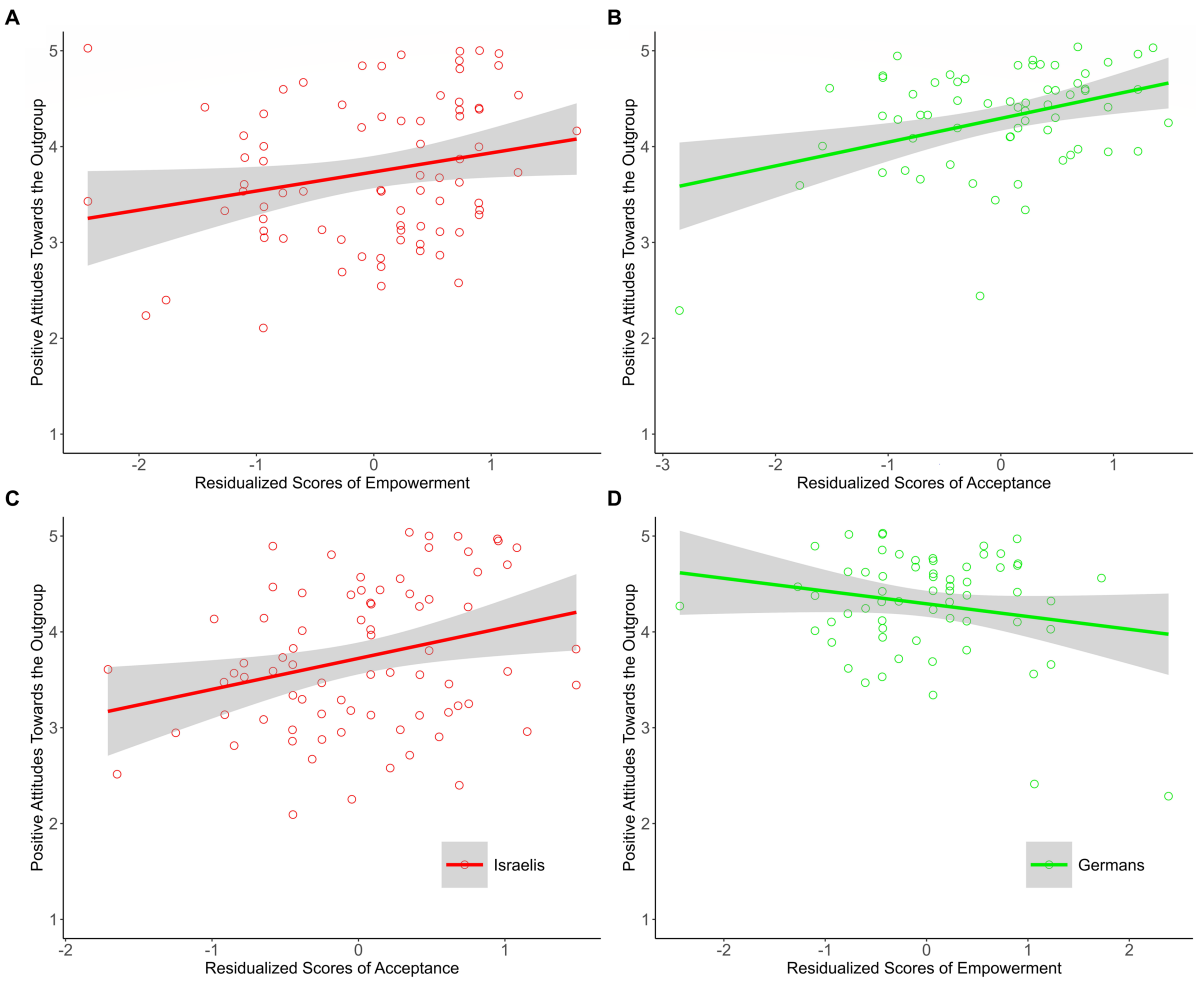
The associations between residualized scores of empowerment and acceptance and positive attitudes toward the outgroup among Israeli and German participants of Study 1. Israeli group (*n* = 77), German group (*n* = 64). The plots for the needs about which we had explicit predictions appear in the upper part of figure **(A,B)**; the plots for the “other’ needs appear in the lower part of figure **(C,D)**. Scatterplots and regression lines with 95% confidence bands for the association between residualized scores of empowerment and acceptance and reconciliatory orientation. Raw data points are jittered.

As expected, prominent messages of empowerment were associated with Israelis’ positive attitudes toward Germans (see Figure 2A). Prominent messages of acceptance, however, were also associated with Israelis’ positive attitudes (see Figure 2C). Among Germans, in line with expectations, prominent messages of acceptance, were associated with positive attitudes toward Israelis; messages of empowerment were not (see Figures 2B,D).

##### Keeping in touch with outgroup members

**Figure 3:**
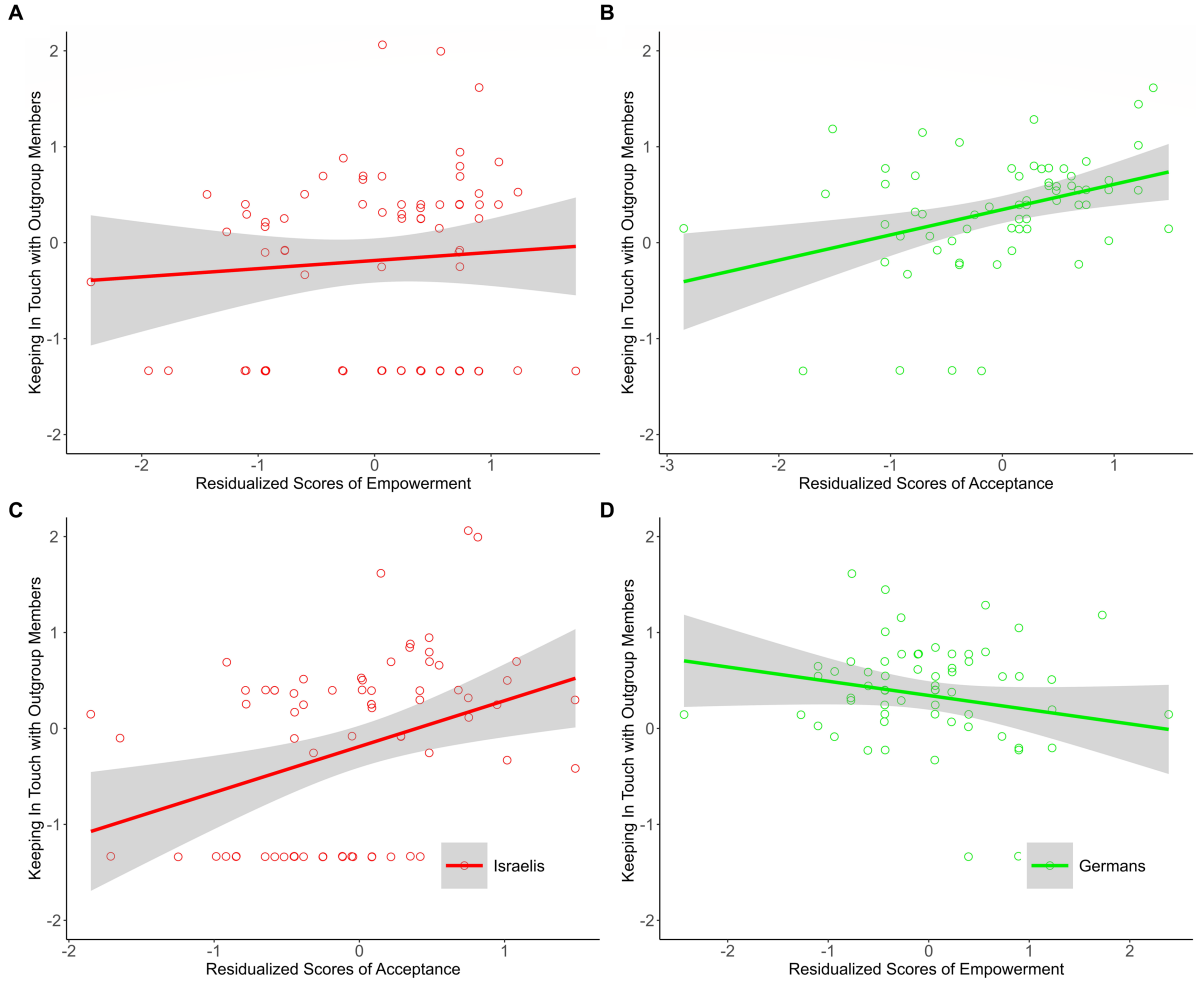
The associations between residualized scores of empowerment and acceptance and keeping in touch with outgroup members among Israeli and German participants of Study 1. Israeli group (*n* = 78), German group (*n* = 64). The plots for the needs about which we had explicit predictions appear in the upper part of figure **(A,B)**; the plots for the ‘other’ needs appear in the lower part of figure **(C,D)**. Scatterplots and regression lines with 95% confidence bands for the association between residualized scores of empowerment and acceptance and reconciliatory orientation. Raw data points are jittered.

Among Israelis, inconsistent with our prediction, prominent messages of acceptance — but not of empowerment — were associated with keeping in touch with Germans following the program (see Figures 3A,C). Among Germans, in line with expectations, prominent messages of acceptance (but not of empowerment) were associated with keeping in touch with Israelis following the program (see Figures 3B,D).

#### Robustness checks

Two robustness checks reported in the Supplemental materials suggest that our conclusions (a) do not depend on extreme observations (outliers) (with the exception of one correlation, between empowerment and positive attitudes toward the outgroup among Israelis, that became marginal; *p* = 0.06), and (b) persist while controlling for background variables (e.g., program duration) that may potentially influence program satisfaction.

#### Responses to open-ended questions

Readers who are interested in “hearing” the participants’ own words are kindly referred to the Supplemental materials with their responses to the open-ended questions. Excerpts by Israeli participants include: (a) “During our time in Germany, we were in a concentration camp. We Israelis and Germans both had a hard time processing it. Walking with the Israeli flag made me feel like a part of my country and grateful Israel exists,” and (b) “At the Ghetto Fighters’ House Museum, our Israeli guide told the story of her parents during the Holocaust and the establishment of Israel. Her story was moving. Germans were moved by her story too.”

Excerpts by German participants include: (a) “All of us stood silent at that moment, remembering the victims together. [...] Germans and Israelis understood we are all human beings and cannot be held accountable for the actions our ancestors committed,” and (b) “Germans and Jews both had a hard time in the concentration camp, but luckily no groups formed, and both Israelis and Germans were there for each other.”

### Discussion

The results of Study 1 generally, but not fully, supported the hypotheses derived from the needs-based model. As expected, Germans’ feelings of acceptance by Israelis during discussions about the Holocaust were associated with their satisfaction with the program (supporting *H*_1b_), as well as with their positive attitudes toward Israelis (supporting *H*_2b_) and keeping in touch with Israelis following the program (supporting *H*_3b_). These findings highlight the importance of historical perpetrator group members’ need for acceptance when discussing the transgression with members of the victim group.

Interestingly, German participants’ feeling that the victim group was empowered in discussions about the Holocaust was associated with *less* program satisfaction. A possible reason for this finding, which is not derived from the needs-based model, maybe that an important component of the empowerment of the Israeli group is the message that the Holocaust will always be a significant part of Jews’ and Germans’ identities. Such a message stands in contrast with historical perpetrator group members’ (e.g., White Americans, Ditlmann et al., 2014) wish to move on from the historical transgression and focus on the present and future. Indeed, many are angry that the crimes of the Holocaust are still held against their group (Hagemann and Nathanson, 2015; Hestermann et al., 2022). Therefore, perhaps discussions in which a highly prominent message was that the Holocaust should always be a part of their group’s identity could lead to Germans’ lower program satisfaction.

Among Israelis, as expected, feelings of empowerment by Germans during discussions about the Holocaust were associated with program satisfaction (supporting *H*_1a_) and positive attitudes toward Germans (supporting *H*_2a_). Positive attitudes toward Germans were also associated with being accepted by Germans and, unexpectedly, keeping in touch with Germans following the program was associated with being accepted, rather than empowered, by them (inconsistent with *H*_3a_). A possible explanation for this unexpected finding is that in the immediate context of the program, Israelis’ victim identity was highly salient, and therefore their need for empowerment, rather than acceptance, predicted program satisfaction. In some broader contexts outside of the program, for example in heated online debates about Israel’s settlement policy, Israelis are often perceived as perpetrators by Germans (Mendel, 2022). Under such circumstances, Israelis’ need for acceptance, rather than empowerment, may play a more crucial role in determining their relations with Germans.

Another interpretation of the results is that Germans’ discomfort with Israeli’s expressions of empowerment is a manifestation of antisemitism. Given the nature of the program and youths’ participants motivation for signing up this is a less plausible explanation. Notably, even if such discussions are not comfortable for German program participants, they are important for Israeli participants, which is, of course, an important reason for including them in the program. Future research should examine the why of the finding and how programs can address it.

Admittedly, the Israeli-Palestinian conflict was discussed during exchange programs^5^. However, based on the understanding that contemporary Antisemitism often manifests as hostility toward Israel (Cohen et al., 2009), one primary objective of the youth exchange programs between Germany and Israel is to eradicate anti-Israel sentiments (Mähler et al., 2015). Therefore, it is likely that Israel’s moral condemnation was lower within the context of the program than in the broader context outside of it, especially the context of social network platforms through which participants communicated and kept in touch with outgroup friends after the program had ended. In such platforms, the Israeli–Palestinian conflict draws much attention (Evans, 2016), and Israel’s actions toward Palestinians are heavily criticized (Seo, 2014). As a result, Israeli participants’ feelings that their ingroup was morally and socially accepted by Germans may be especially crucial for determining their tendency to keep in touch with outgroup members. Study 2 aimed to shed light on this possible explanation.

A limitation of Study 1 is that some of the participants took part in the same exchange program, which means that our observations were not independent of each other. A multilevel cluster analysis was therefore required to take into account the similarities between participants of the same program (Raudenbush and Bryk, 2002). However, since fewer than 20% of the participants could be classified into groups containing at least four participants, which is a requirement for conducting a multilevel cluster analysis (McNeish, 2014), we refrained from performing this analysis. As noted earlier, many participants answered the question “In which program did you participate?” in a way that prevented identification of the program (e.g., “from school”). In many cases, the program was also described too generally (e.g., “the Scouts”) although the Scouts operate several different programs. To overcome this limitation, future studies may ask participants about the programs they participated in using a closed-ended rather than an open-ended question.

## Study 2

Study 2 examined whether Israeli Jews’ perceptions of messages of empowerment and acceptance by Germans as conciliatory depend on whether these messages are conveyed in the social context of the Holocaust (in which their ingroup’s victim role is salient) or the Israeli-Palestinian conflict (in which their ingroup’s perpetrator role is salient). In the context of the Israeli–Palestinian conflict, Israelis often perceive themselves as victims of injustice and aggression [Bar-Tal et al., 2009; see also Hirschberger et al., 2016 for Israelis’ experience of existential threat and fear of annihilation by Palestinians]. This is also true for young Israelis (Pilecki and Hammack, 2014). Nevertheless, Israelis also experience a threat to their moral image as they are aware of the criticism directed at Israel for its actions against the Palestinians, and groups accused of immoral conduct face the threat of social exclusion (Tavuchis, 1991). Thus, when Israelis are accused of unjustly harming Palestinians, being morally accepted by Germans could be psychologically meaningful in alleviating the threat of moral condemnation and social exclusion.

To test Study 2’s prediction, Israeli Jewish participants were randomly assigned to read information about either Antisemitic propaganda during the Nazi era (Holocaust condition) or about the condemnation of Israeli policy toward Palestinians (Israeli-Palestinian conflict condition). They were then exposed to both an empowering message and an accepting message, allegedly conveyed by two German parliament members in response to this information. Our prediction (*H*_4_) that Israeli Jews would perceive the empowering (vs. the accepting) message to be more conciliatory in the Holocaust condition than in the Israeli-Palestinian conflict condition was preregistered on https://aspredicted.org/8KB_C9D.

### Method

#### Participants

An *a priori* power analysis using the G*Power calculator (Faul et al., 2009) indicated that to detect a small-to-medium effect size of *d* = 0.3 [based on Cohen’s (1988) guidelines] with a significance level of 5% and a power of 80%, we needed a sample of at least 278 participants. Participants were recruited to take part in an online study by an Israeli survey company in October 2022.

After the exclusion of participants who failed the Instructional Manipulation Check (IMC; Oppenheimer et al., 2009) based on the exclusion criteria specified in the preregistration, our sample included 293 Israelis (153 men, 140 women). All participants (*M*_age_ = 38.35, *SD* = 11.37, range = 25 to 78) were Israeli Jews. In terms of educational level, 7.6% of participants had completed elementary education (i.e., less than 12 years of formal education), 38.9% had completed high school education, and 53.6% had completed higher education (ranging from undergraduate degree to PhD); 5.5% of the participants were current students. Most participants identified as rightists (57.7%); the rest were on the left (23.2%) or center (19.1%) of the political spectrum. Data, protocol, and Supplemental Materials are available on the OSF: https://osf.io/cukfw/?view_only=700728c9cae84b32a54e1e9926a11080.

#### Procedure

The study was presented as research on reactions to different types of messages in the context of contemporary relations between Jews and Germans. Participants were randomly assigned to the two context conditions. In the Holocaust condition, participants were exposed to posters of Nazi Antisemitic propaganda ostensibly presented in an exhibition held at the Yad Vashem Museum (the World Holocaust Remembrance Center). These Nazi posters illustrated the differences between the “inferior Jewish race” and the “superior Aryan race” and the danger of “contamination” faced by the “Aryans”. In the Israeli-Palestinian conflict condition, participants read posts about the hardship faced by Palestinian children under the Israeli occupation, ostensibly posted by pro-Palestinian activists on the Twitter account of Olaf Scholz, the Chancellor of Germany (see Supplemental materials for more information about the images). It is important to note that the factual occurrences that are the focus of the materials presented in Study 2 cannot and should not be compared. However, according to the needs-based model, some aspects of individual experience when encountering such materials, namely the threat of social exclusion due to moral exclusion may have similarities regardless of the transgressions’ severity, duration and so forth.

These particular platforms (a museum exhibition for the Holocaust condition, a social network post for the Israeli Palestinian condition) were chosen to simulate the real-life conditions under which Israelis’ may encounter materials that make salient their social role as victims or perpetrators. Notably, the goal of Study 2 was not to isolate the effect of platform from the effect of social role saliency, but rather to establish the ideal conditions (consisting of both role saliency and platform) for testing the plausibility of our account for the unexpected finding of Study 1 (see Aydin et al., 2019 for a similar approach).

Next, participants were presented with two excerpts from texts, which were allegedly written by two German parliament members (‘Max Dittmann’ and ‘Peter Schultis’) either in Yad Vashem’s visitor book (Holocaust condition) or on Twitter (Israeli Palestinian conflict condition). One excerpt conveyed an empowering message (“…the Jewish people contributed and continue to contribute to advancing humanity in science, medicine, culture, and all areas of life”), whereas the other excerpt conveyed an accepting message (“…there is a deep friendship between the German people and the Jewish people, and this is not expected to change”). The order of the messages (whether the first message was empowering or accepting) and the name of the German parliament member who conveyed them (whether Max Dittmann conveyed the empowering message and Peter Schultis conveyed the accepting message or the other way around) were counterbalanced.

Manipulation checks verified that participants correctly understood the content of the messages. Three items assessed the extent to which participants understood the message as empowering (e.g., “Which message expresses more respect toward the Jewish people?”; α = 0.801), using a 6-point scale (ranging from 1 = *definitely the message from Max Dittmann* to 6 = *definitely the message from Peter Schultis*). Participants’ responses were averaged, such that higher scores indicated understanding the empowering message as more empowering than the accepting message. Correspondingly, using the same 6-point scale, three items assessed the extent to which participants understood the message as accepting (e.g., “Which message expresses greater friendship between the Jewish and German people?”; α = 0.935). Participants’ responses were averaged, such that higher scores indicated understanding the accepting message as more accepting than the empowering message.

Participants’ *perceptions of the empowering (*vs. *the accepting) message as conciliatory* were assessed using four items; e.g., “Which message is more appeasing?”; “Which message is likely to make Jews feel welcomed in Germany (e.g., can move there for studies or professional training?”; α = 0.865), using 6-point scale (ranging from 1 = *definitely the message from Max Dittmann* to 6 = *definitely the message from Peter Schultis*). Participants’ responses were averaged such that higher scores indicated perceptions of the empowering (rather than the accepting) message as more conciliatory. Upon completion, participants reported their demographics and whether they encountered technical problems during the study. They were then thanked and debriefed.

### Results and discussion

#### Manipulation checks

To verify that participants understood the content of the messages as intended, we conducted one-sample *t*-tests. Participants’ understanding of the empowering message as empowering (i.e., conveying respect and appreciation of their ingroup) was significantly above the scale’s midpoint, *M* = 4.6, *SD* = 1.2, *t*(292) = 16.22, *p* < 0.001, *d* = 0.95 (indicating that it was perceived as more empowering than the accepting message). Correspondingly, participants’ understanding of the accepting message as accepting (i.e., expressing friendship between the two groups), *M* = 4.7, *SD* = 1.4, was significantly above the scale’s midpoint, *t*(292) = 15.63, *p* < 0.001, *d* = 0.91 (indicating that it was perceived as more accepting than the empowering message). Thus, the content of the messages was manipulated successfully.

#### Perceptions of the empowering message as conciliatory

In line with prediction, participants’ perception of the empowering (vs. the accepting) message as conciliatory was higher in the Holocaust condition, *M* = 4.2, *SD* = 1.3, than in the Israeli-Palestinian conflict condition, *M* = 3.0, *SD* = 1.3; *t*(291) = 7.63, *p* < 0.001, *d* = 0.89. Put differently, supporting *H*_4_, the empowering message was perceived as more conciliatory in the context of the Holocaust than in the context of the Israeli-Palestinian conflict, whereas the accepting message was perceived as more conciliatory in the Israeli-Palestinian than in the Holocaust context. Taken together, the results of Study 2 (including additional exploratory analyses, which are presented in the Supplemental materials) supported our theorizing that Israeli Jews’ response to being empowered or accepted by Germans depends on whether the Holocaust or the Israeli-Palestinian conflict is more contextually salient. These results support the plausibility of our account for the unexpected finding of Study 1 that feeling accepted, rather than empowered, associated with the tendency of Israeli participants of youth exchange programs to keep in touch with German participants.

#### A conceptual replication of Study 2

The needs-based model of reconciliation subsumes perpetrator group members’ needs for restoration of moral identity and acceptance together, based on the assumption that moral transgressors face the threat of social exclusion (Shnabel et al., 2023). The boycott of Russia in response to its invasion to Ukraine may serve as a recent real-life example of the link between (im)morality and exclusion. Other theoretical perspectives, however, stress the unique importance that group members ascribe to their moral identity, highlighting the distinction of morality from other identity dimensions (see Brambilla et al., 2021). To address the criticism that the model might conflate the needs for morality and acceptance, we conceptually replicated Study 2 while replacing the accepting message with a morally affirming message. The conceptual replication study (*N* = 296) used the same design and method as Study 2 with only one change: the message stressing the friendship between the German and Jewish people was replaced with a message that affirmed the Jews’ moral identity (“…the Jewish people stood out throughout history, and continues to stand out even today, ethically and morally”). The findings of Study 2 were replicated with no change in statistical conclusions (see Supplemental Materials). Data, protocol, and Supplemental Materials are available on the OSF: https://osf.io/cukfw/?view_only=700728c9cae84b32a54e1e9926a11080.

## General discussion

Two studies examined hypotheses derived from the needs-based model of reconciliation in the context of contemporary relations between Israeli Jews and Germans. Study 1, the main study was a field study that tested, among Israeli Jewish and German participants of youth exchange programs, whether and how feelings of empowerment and acceptance in discussions about the Holocaust translated into satisfaction with the program and reconciliatory orientation toward the outgroup. Study 2 examined a novel hypothesis (based on the unexpected findings observed in Study 1) according to which the needs of historical victim group members vis-à-vis the historical perpetrator group depend on the social context.

The results of Study 1 generally supported our hypotheses. With regards to program satisfaction, in line with predictions, Israeli participants’ feeling that their ingroup was empowered in these discussions was associated with greater satisfaction. Correspondingly, German participants’ feeling that their ingroup received moral-social acceptance in these discussions was associated with their greater satisfaction. That German and Israeli survey participants came from diverse regions in their home countries, and took part in diverse programs (exchange between twin cities, sports clubs, and so forth) that were delivered in different years, strengthens our belief that the results are not unique to specific circumstances (e.g., reflect the situation in one particular exchange program). Rather, they seem to reflect generalizable links between need satisfaction and program satisfaction—a conclusion that is practically important for program leaders.

As for reconciliatory orientation, in line with expectations, German participants’ feeling that their ingroup was accepted by the Israeli participants was associated with more positive attitudes toward Israelis and keeping in touch with Israelis following the program. Israeli participants’ positive attitudes toward Germans were associated not only with the feeling that their ingroup was empowered during the discussions but also with feelings of acceptance. Moreover, opposite to expectations, Israeli participants’ keeping in touch with Germans following the program was not associated with their feelings of empowerment, but rather with their feeling that their ingroup was accepted by Germans.

Study 2 tested a possible explanation for the unexpected finding among Israeli participants—namely, that outside the immediate context of the youth exchange program (e.g., in the context of social networks) Israelis’ social identity as perpetrators vis-à-vis the Palestinians may be more salient than their social identity as victims vis-à-vis Germans, raising their need for moral acceptance (rather than empowerment). Supporting this possibility, Study 2 revealed that Israeli Jewish participants’ perceptions of expressions of empowerment or acceptance by German representatives as conciliatory depended on the context in which these expressions were conveyed. In the context of the Holocaust, consistent with previous findings (Shnabel et al., 2009), Israeli Jews perceived the empowering message, which conveyed respect and appreciation of the Jewish people, as more conciliatory than the accepting message. In the context of the Israeli-Palestinian conflict, by contrast, the opposite pattern was observed such that the accepting message, which conveyed friendship and commitment, was perceived as more conciliatory.

Thus, even though the Holocaust continues to serve as a point of reference for Israeli Jews when examining their relationship with Germans (Hestermann et al., 2022), when the context of the Israeli-Palestinian conflict becomes salient they seem to experience the need to feel accepted by Germans. Besides shedding light on the unexpected finding of Study 1, the results also provide an especially powerful demonstration of the fluidity of social identity, as assumed by self-categorization theory (Turner et al., 1987), by suggesting that group members may experience identity needs associated with perpetration even vis-à-vis members of the group responsible for their chosen trauma (Volkan, 2021). This fluidity of social identity underscores the increased complexity when moving from the lab to the field. Whereas it is possible to ‘isolate’ the social context of interest in the lab, such control over the setting is not possible in the field.

Indeed, in recent years there is a growing understanding that to establish social psychological models it is important to test their generalizability across various contexts and using diverse operationalizations [Simons et al., 2017; see also Eronen and Bringmann (2021) call to gather more robust evidence for phenomena that are already ‘discovered’ in psychology]. This was the goal of Study 1, which tested the needs-based model for the first time in the context of youth exchange programs [see Hanel and Vione (2016) for the importance of using non-student samples] while using new operationalizations of need satisfaction (measured as the prominence of empowering and accepting messages in discussions about the Holocaust), reconciliatory orientation (measured as both explicit outgroup attitudes and actual contact maintenance with outgroup members).

Taken together, the findings of Study 1 highlight its relevance as a conceptual framework for leaders of exchange programs between members of former adversarial groups; the findings of Study 2 point to the importance of attending to the particular and dynamic social context when applying the model’s insights to designing and leading such programs.

### Limitations and future directions

One limitation of Study 1 is its cross-sectional design, which limits causal inference. The logical chronological order is that need satisfaction in discussions about the Holocaust, which occurred during the program, should precede subsequent program satisfaction and reconciliatory orientation (which includes post-program behaviors, such as keeping in touch with outgroup members). Nevertheless, we acknowledge that the opposite direction is also possible. For example, maintaining frequent and positive contact with outgroup members may ‘color’ participants’ memory of the discussions during the programs, making them remember a higher degree of need satisfaction in retrospect. Moreover, in the current study, we could not partial out the effect of preexisting reconciliatory orientation on the variables of interest. Future research may use a longitudinal design, which will allow to examine changes over time, prior to and after the program, in participants’ experience of need satisfaction, program satisfaction and reconciliatory orientation. Furthermore, using experimental design, future research may manipulate the level of need satisfaction by modifying the relative composition of activities within the exchange program that promote mutual acceptance through friendship formation (e.g., hiking and outdoor sports activities) or empowerment through acknowledgment of past atrocities (e.g., visits to remembrance centers). In the present study, we focused on developing strong partnerships with stakeholders (i.e., our ConAct partners), as establishing credibility with external stakeholders is crucial to making experiments in the field feasible (Lewis, 2021). However, we hope to conduct a controlled randomized field experiment in the future.

A second limitation of Study 1 is that participation in the survey was not mandatory for all program participants, raising the threat of selection bias (Campbell, 1957). Possibly, German and Israeli participants who chose to complete the survey were especially satisfied with the program (and therefore felt obligated to share their appreciation for it). That the distribution of program satisfaction was negatively skewed (−0.85; Shapiro–Wilk *W* = 0.91, *p* < 0.001) is consistent with this possibility. However, because the goal of Study 1 was to evaluate the associations between the constructs of interest, rather than to estimate parameters in the populations of interest (e.g., determining whether overall participants felt satisfied with the programs, or evaluating the programs’ effectiveness), the threat of selection-bias on the validity of our conclusions might be smaller than seems at first glance. Nevertheless, we acknowledge that our conclusions are limited to individuals who were relatively satisfied with the program. Future research may collect data from participants on their satisfaction with the program at the end of the program before they return home, encouraging greater participation, including those who experienced lower satisfaction from the program.

As for Study 2, one limitation is in testing the hypotheses in an artificial rather than natural environment. For example, rather than examining participants’ actual responses (such as sharing or liking) to real posts on Twitter, participants were asked about their perceptions of artificial messages allegedly posted on this social network. Like other lab or online experiments in social psychology, doing so allowed causal inference, while compromising ecological validity (Cialdini, 2009). A second limitation of Study 2 is that participants were not Israeli Jews who participated in German-Israeli youth exchange programs, but rather older Israelis who represent the general population of Jewish citizens of Israel but - like program participants - center-left and left leaning in terms of political ideology. Using different populations is often an inevitable part of integrating basic and applied research (Lewis, 2021), because applied research focuses on components of external validity (such as representativeness in samples) whereas basic research focuses on components of internal validity (such as establishing causality). Notably, however, according to social identity theory, people belong to a variety of social groups, but the saliency of a specific identity is determined by the context; when salient, the relevant social identity, including how others perceive it, shapes behavior by the norms, values, and beliefs that define it (Reicher, 2004). Therefore, despite the fact that participants in youth exchange programs possess some distinctive characteristics that are not shared by the Israeli Jews from the general population (whom we were able to recruit in Study 2), it is plausible that participants in both studies had similar responses (in terms of experienced needs) to social contexts where their Israeli or Jewish identity was psychologically threatened. Both limitations can be addressed in future research that would follow the social network activity of Israeli participants of exchange programs vis-à-vis their German counterparts. Since social networks play a central role in young people’s social lives (Rideout et al., 2022), monitoring their activities on such sites can provide insight into social psychological processes that influence intergroup relations between former adversaries.

From a broader perspective, a general limitation of the needs-based model of reconciliation is that it applies to historical conflicts where there is a high social consensus regarding victim and perpetrator roles, as it is the case for the Holocaust, and for example, relations between White and Indigenous/First Nation people in Australia or Canada referring to the residential school systems. The model applies less well in cases where the social roles of perpetrators and victims are highly contested, such as the Israel/Palestine conflict. And while it is interesting theoretically that - even in such a contested context - participants temporarily experience the psychological need the model predicts, this is likely going to shift quickly as the immediate social context changes, and as such maybe less informative for the design of dialogue programs.

Finally, the present study was based on the social identity perspective, according to which individuals may identify as perpetrators or victims only by their social identity (i.e., by their identification with a particular group), regardless of their personal involvement in the events (Tajfel and Turner, 1979). For example, in his analysis of the intergenerational transmission of chosen trauma, Volkan (2021) suggests that Milošević’s quest to bring Prince Lazar’s remains to Kosovo for burial evoked feelings of victimhood following events 600 years earlier. It would, however, be interesting for future research to examine whether and how direct personal involvement in the conflict affects the need for empowerment and acceptance, and the willingness to reconcile with the outgroup.

### Practical implications

The insights gained through the present studies suggest that intergroup dialogue interventions should be designed with awareness of the differential psychological needs of members of historical victim vs. perpetrator groups, as well as the psychological needs resulting from the current state of intergroup relations. The leaders of such interventions should also be aware of the risk that addressing one group’s needs might come at the expense of addressing the other group’s needs, and try to overcome it. Additionally, it is important to note that empowerment and acceptance are multifaceted concepts that can mean different things depending on the context (Shnabel et al., 2023). For example, since Turkey denies the Armenian genocide (Bilali, 2013), Turks taking responsibility for the historical injustice can be conceptualized as empowering the Armenians. However, when culpability has already been acknowledged (e.g., Canada’s apology to the First Nations peoples), further steps are needed to empower the victim group, including compensation and redistribution policies [see Wohl et al.’s (2011) ‘staircase model,’ which describes the sequential steps required for empowering the victim group as part of the road to reconciliation].

Acceptance can similarly mean different things in different contexts. For example, the post-Apartheid concept of ‘rainbow nation’ (i.e., the idea of a common national identity for all South Africans) can be perceived as conveying acceptance by Black, Colored, and Indian South Africans toward White South Africans (Evans, 2010). This concept, however, is irrelevant to the context of German–Jewish relations. Broadly speaking, the assumption underlying our theoretical framework is that members of historical perpetrator groups experience the need for “re-humanization,” because their ingroup is perceived as purely evil [which may be conceptualized as a form of dehumanization; Formanowicz et al., 2023; see also Staub (2006) discussion of the Hutu’s need for re-humanization following the Tutsi genocide]. Expressions of acceptance, which re-humanize the perpetrator group and its members, can be manifested in different ways. These include politicized forms of acceptance, such as solidarity with the perpetrator group and empathetic acknowledgment of the circumstances that led to its violence. Non-politicized forms of acceptance include acknowledgment of the humanity of members of this group, regardless of their ingroup’s behavior, or willingness to be friends with its members. Thus, the present research does not offer concrete ‘recipes’ for designing interventions to promote reconciliation. Rather, it offers general insights about the different needs of members of historical victim and perpetrator groups and how their satisfaction can serve to facilitate a constructive dialogue.

In the present research, we measured (Study 1) or manipulated (Study 2) non-politicized forms of acceptance, such as commitment to each other as human beings regardless of our group affiliation (Study 1), or highlighting the friendship between the groups (Study 2). The responses to the open-ended questions in Study 1 (see Supplemental materials) also referred only to non-politicized forms of acceptance, such as interpersonal friendship across group lines. We admit, however, that to achieve reconciliation it would be interesting to examine to achieve reconciliation in future research whether expressions of politicized (vs. non-politicized) acceptance have a stronger effect on group members who receive it.

## Conclusion

Dialogic processes between members of historically victim and perpetrator groups do not necessarily bring the groups closer together and might even increase the hostility between them (Gergen et al., 2001; see also Bilewicz, 2007; Maoz et al., 2007). It is therefore important to design intergroup dialogue interventions in ways that promote reconciliation, rather than further fuel the conflict (e.g., Bilewicz and Jaworska, 2013). We hope that the insights gained through the present studies will be useful for practitioners who engage in efforts to promote a transformative dialogue through people-to-people interventions—between Germans and Israelis, as well as other groups dealing with a historical conflict.

## Data availability statement

The raw data supporting the conclusions of this article will be made available by the authors, without undue reservation.

## Ethics statement

The studies involving humans were approved by The Ethics Committee of Tel-Aviv University. The studies were conducted in accordance with the local legislation and institutional requirements. Written informed consent for participation in this study was provided by the participants or their legal guardians/next of kin. Participants in study 2 were debriefed about the full purpose of the research.

## Author contributions

NS and RD conceptualized the main idea of Study 1. GP-M and NS conceptualized the main idea of Study 2. GP-M designed and analyzed the results of Studies 1 and 2 with support from NS, Study 1 with support from RD. GP-M wrote the manuscript with support from NS and RD. All authors provided critical feedback and helped shape the manuscript.

## Footnotes

^1^As mentioned before, the model is about perceptions and makes no moral claims, nor does it compare incidences in terms of their historical gravity.

^2^For details and a discussion of the term "chosen" please check the reference. Importantly, the term does not imply the undermining of the suffering, injustice, and trauma done to the victim group.

^3^Importantly, the research was conceived and conducted before the Hamas attacks on Israel on October 7th, 2023. The psychological impact of these attacks resulted in collective trauma (Hirschberger, 2018), and may contribute to the development of a chronic empowerment need also in the context of the Israel Palestinian context.

^4^While, in principle, this item could also imply support for the two-state as opposed to the one-state solution to the Israeli-Palestinian conflict - we believe that in the present context (i.e., when the Holocaust is salient) it was interpreted as a recognition of Jews’ right for statehood (see Adwan and Bar-On, 2004, for Jewish people’s right for existence through statehood as a core component of the Israeli narrative).

^5^Five items using a 5-point scale (1 = *not part of my program* to 6 = *extremely central*) assessed the centrality of different program components, including activities related to the Holocaust (e.g., visiting memorial sites) and to the Israeli-Palestinian conflict (e.g., visiting the separation barrier). A paired samples *t*-test revealed that the activities related to the Holocaust (*M* = 3.7, *SD* = 1.1) were more central during the programs than activities related to the Israeli-Palestinian conflict (*M* = 2.4, *SD* = 1.2), *t*(166) = 12.03, *p* < 0.001, *d* = 0.93.
